# Statin as anti-cancer therapy in autochthonous T-lymphomas expressing stabilized gain-of-function mutant p53 proteins

**DOI:** 10.1038/s41419-020-2466-4

**Published:** 2020-04-24

**Authors:** Karis Tutuska, Laura Parrilla-Monge, Erica Di Cesare, Alice Nemajerova, Ute M. Moll

**Affiliations:** Department of Pathology, Stony Brook University, Stony Brook, NY USA

**Keywords:** Cancer models, Cancer models, Cancer therapy, Cancer therapy

## Abstract

An important component of missense mutant p53 gain-of-function (mutp53 GOF) activities is the ability of stabilized mutp53 proteins to upregulate the mevalonate pathway, providing a rationale for exploring the statin family of HMG-CoA reductase inhibitors as anticancer agents in mutp53 tumors. In this small exploratory study we report on the effects of statin treatment in autochthonous mouse models of clinically advanced T-cell lymphoma expressing two different GOF mutp53 alleles. We find that Rosuvastatin monotherapy shows a modest, p53 allele-selective and transient anti-tumor effect in autochthonous T-lymphomas expressing the p53 R248Q DNA contact mutant, but not in tumors expressing the p53 R172H conformational mutant. p53 null mice also do not benefit. In vitro statin sensitivity is not a strong predictor for in vivo sensitivity, while subcutaneous allografts are. Future explorations of statins in combination therapies are justified to improve its anti-tumor effects and to better define the most statin-sensitive alleles and tumor types among mutp53-stabilized cancers.

## Introduction

Statins have long been suspected to have antitumor activity. Some epidemiological evidence correlates coinciding statin use with reduced mortality from sporadic cancers including colorectal, breast, lung, prostate, and kidney cancers^1–9^. This is supported by preclinical studies that show that statins directly inhibit e.g. prostate and pancreatic cancer development and progression in cell- and animal-based models^2,3,10,11^. However, other studies failed to find this association^12,13^.

TP53 is the most commonly mutated gene in human cancer. The majority of mutations are p53 missense mutations (mutp53) that have not only lost their tumor suppressor function, but often acquire broad oncogenic driver gain-of-function (GOF) activities promoting cancer progression, EMT, invasion, metabolism, metastasis, and chemoresistance^14^. Mutp53 stabilization is a prerequisite for GOF. Tumors with stabilized GOF mutp53 exhibit dependency on continued expression of high levels of mutp53 protein for tumor maintenance, invasion and metastasis. Genetic or pharmacological depletion of GOF mutp53 results in tumor regression or stagnation in spontaneous T-lymphoma and carcinogen-induced colorectal carcinoma in autochthonous mouse models. This translates to significant gains in survival^15,16^ and makes degradation of mutp53 a promising drug target for anti-cancer therapy.

One avenue for downregulating GOF mutp53 is to target the heat shock protein 90 chaperone machinery composed of the Hsp90 ATPase, co-chaperones and adapter proteins (the HSP90 complex)^15,16^. HSP90 is constitutively and ubiquitously upregulated in transformed versus normal cells since it bestows resistance to proteotoxic stress and protects against tumor cell death by preventing aggregation and supporting proper folding of conformationally aberrant oncoproteins including mutp53^17,18^. The intrinsically unstable mutp53 proteins accumulate specifically in cancer but not in normal cells to high levels because they are protected by the HSP90 machinery from degradation by their E3 ubiquitin ligases Mdm2 and CHIP by engaging in stabile complexes with HSP90^19^. Hsp90 and mutp53 also exist in a positive feed-forward loop whereby mutp53 activates the heat shock factor 1 (HSF1) master transcription factor, which in turn induces Hsp90 (as well as other heat shock proteins including Hsp70, Hsp40 DNAJA1) which stabilize mutp53^20^. In mutp53 knockin mice small molecule inhibitors targeting Hsp90 (e.g. 17AAG, Ganetespib) and/or its positive regulator HDAC6 (e.g. SAHA) degrade stabilized mutp53 in tumor tissues and markedly extend survival in a mutp53-specific manner, since they have no benefit in p53 null mice^15,16^.

Likewise, the Hsp40 chaperone isoform DNAJA1, in particular the farnesylated form^11^, in conjunction with Mevalonate-5-phosphate (MVP), an intermediate metabolite of the mevalonate pathway, also stabilizes mutp53 by sheltering it from CHIP-mediated ubiquitination and proteasomal degradation^21^. The mevalonate pathway is responsible for de novo synthesis of endogenous cholesterol and many non-sterol isoprenoid lipid derivatives such as geranyl-geranyl-PP for protein prenylation including protein farnesylation. Protein prenylation facilitates cell membrane insertion and membrane interactions, promotes protein‐protein interactions, and can affect protein turnover^22^. Statins potently downregulate this pathway by inhibiting its rate-limiting enzyme HMG-CoA reductase (HMGCR), thereby also reducing downstream MVP levels^23^. Statins were found to reduce protein levels of conformational p53 mutants (R156P, Y157F, R175H, and Y220C) in human and murine cancer cell lines by liberating mutp53 from its protective interaction with DNAJA1 and enabling CHIP-mediated degradation. This was associated with impaired growth in cultured cells and in xenografts^21^.

Statins also reactivate the Mdm2-dependent degradation of mutp53 by disrupting the protective mutp53-Hsp90 complex via two mechanisms. First, statins directly inhibit HDAC6 activity, leading to Hsp90 acetylation and inactivation causing mutp53 destabilization^24,25^. Second, RhoA-GTPases, which require mevalonate pathway-dependent geranyl-geranylation-mediated anchoring in the cell membrane to transduce mechanical signals from the extracellular environment via the actin cytoskeleton, also contribute to mutp53 stabilization by sustaining the HDAC6/Hsp90-dependent mutp53 accumulation. Thus, by impairing RhoA geranylation, statins also disrupt the mevalonate-RhoA axis of mutp53 accumulation^25^.

While statins were independently identified by two high-throughput small compound screens for mutp53 destabilizers^21,25^, evidence of their preferential cytotoxic effects against mutp53 cancer cells is currently largely limited to in vitro studies. Here, we report the first small exploratory therapeutic study on anticancer statin effects in autochthonous mouse models of GOF mutp53-driven lymphomas at the stage of advanced disease.

## Results

**Fig. 1:**
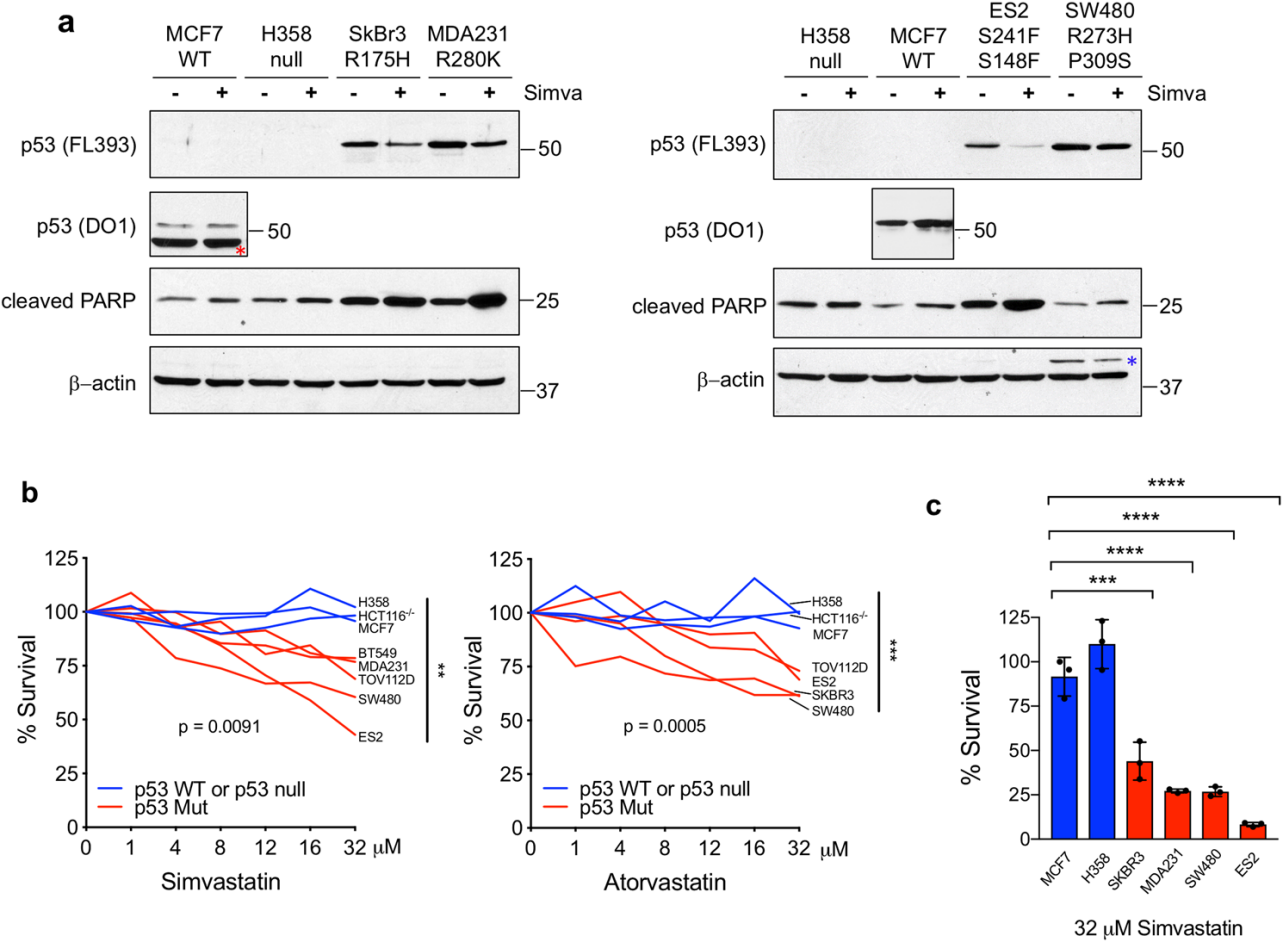
Statins downregulate mutant p53 proteins and exhibit cytotoxic effects in human cancer cell lines. **a** Simvastatin downregulates mutant but not wildtype (WT) p53, while simultaneously upregulating cleaved PARP. Immunoblot analysis of MCF7 (p53 wildtype), H358 (p53 null) and the mutp53 cancer cell lines SkBr3 (p53 R175H), MDA231 (p53 R280K), ES2 (p53 S241F, S148F), and SW480 (p53 R273H, P309S) treated with 32 μM Simvastatin for 48 h. Wildtype p53 levels in MCF7 cells are much lower than mutant levels and were probed separately with DO1 and shorter exposure. β-actin as loading control. Red asterisk indicates persistent β-actin signal from previous blotting of the same membrane, blue asterisk indicates a corresponding persistent p53 signal. This experiment was repeated in three independent experiments with similar results. **b** Survival of mutp53 versus control (WT and p53 null) cell lines in response to Simvastatin and Atorvastatin treatment. Dose response curves after 48 h of the indicated concentrations. CellTiter Blue viability assays of the mutant p53 lines ES2 (p53 S241F, S148F), SW480 (p53 R273H, P309S), TOV112D (p53 R175H), MDA231 (p53 R280K) and BT549 (p53 R249S) and SkBr3 (p53 R175H), compared to wildtype (MCF7) and p53 null (H358 and HCT116−/−) cells. The percent viability at endpoint of all mutant p53 lines was compared to the percent viability at endpoint of p53 null and WT lines at 32 μM Simvastatin. Unpaired Student’s *t*-test, ***p < 0.001, **p < 0.01. Two independent experiments were run with similar results. **c** Survival of mutp53 vs control cell lines after Simvastatin. Cells were treated with 32 µM Simvastatin and live cells only counted at 48 h in Cellometer Cell Counting Chambers. Unpaired Student’s *t*-test, ****p < 0.0001, ***p < 0.001 from three independent experiments. Error bars represent s.d. Two independent experiments were run with similar results.

In a panel of human mutp53 cancer cell lines representing various conformational missense mutations and carcinoma entities, Simvastatin (32 µM for 48 h) downregulated mutp53 and simultaneously upregulated cleaved PARP, albeit to different degrees, in all lines tested, but failed to do so in p53 wildtype (WT) cancer cells (Fig. 1a). Similar results were seen with Atorvastatin (data not shown). Viability assays (CellTiter Blue) confirmed that Simvastatin and Atorvastatin have a dose-dependent cytotoxic effect preferentially on mutp53, while WTp53 and p53 null cells do not respond (Fig. 1b). Moreover, in cell survival assays mutp53 cell lines responded to Simvastatin (32 µM for 48 h) by an up to 12-fold increase in cell death (ES2 cells) compared to WT and p53 null controls (Fig. 1c), confirming previous findings^21,25^. Thus, statins downregulate mutant p53 proteins and exhibit cytotoxic effects in human cancer cell lines.

**Fig. 2:**
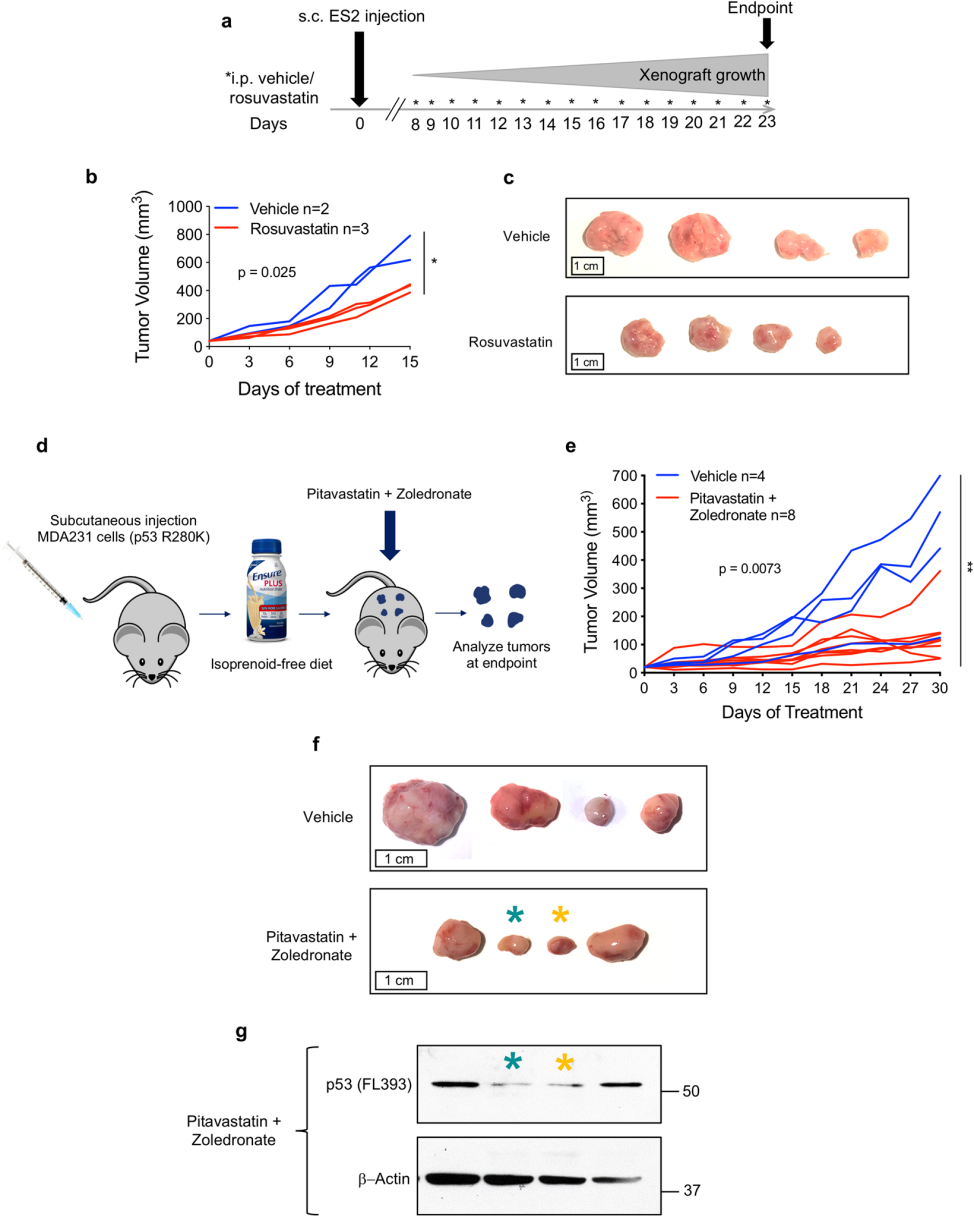
Statins slow tumor growth in human cancer cell xenografts expressing mutp53. **a**–**c** Rosuvastatin attenuates growth in mutp53 subcutaneous xenograft tumors. One million mutp53 (S241F, S148F) ES2 cells each were injected into four dorsal locations of athymic nude mice. When tumors became palpable (1 week after injection), mice were randomized and treated daily with either vehicle or 10 mg/kg Rosuvastatin intraperitoneally. Tumor size was measured by caliper every 3 days. **a** Timeline of the experiment. **b** Quantitation of tumor growth per mouse. Each line represents the average of all four tumors per mouse. Average tumor volumes were normalized to an initial volume of 40 mm^3^. Mean tumor volumes per mouse at endpoint were compared between vehicle-treated and Rosuvastatin-treated mice. Unpaired Student’s *t*-test. *p < 0.05. **c** Representative tumor sizes at endpoint between vehicle and Rosuvastatin-treated groups. **d**–**g** The combination of Pitavastatin + Zoledronic Acid significantly slows tumor growth in mutp53 subcutaneous xenografts. One million mutp53 (R280K) MDA-MB-231 cells each were injected into four dorsal locations of athymic nude mice. Simultaneously, mice were switched to an isoprenoid-free diet where standard mouse chow was removed and replaced with Ensure^R^ plus ad libitum. When tumors became palpable, mice were randomized and treated with either vehicle or 59 mg/kg Pitavastatin orally every 12 h as well as treated with Zoledronic Acid or vehicle intraperitoneally every 3 days. Tumor size was measured by caliper every 3 days. **d** Schema of experiment. **e** Quantitation of tumor growth per mouse. Each line represents one tumor. All tumor measurements were normalized to an initial volume of 20 mm^3^. Individual tumor volumes at endpoint were compared between tumors from vehicle-treated mice and tumors from drug-treated mice. Unpaired Student’s *t*-test. **p < 0.01. **f** Representative tumor sizes at endpoint between vehicle and treated groups. **g** The favorable response to statin + zoledronate treatment is correlated with lower levels of mutp53 in tumors shown in **f**. Teal and yellow asterisks indicate which protein bands in the immunoblot (**g**) respond to which tumors in **f**. The two drug-treated tumors with the lowest levels of mutp53 were some of the smallest tumors at endpoint. Tumor lysates were immunoblotted twice with similar results (technical replicates).

To determine the therapeutic effects of statins in vivo, we next treated immunocompromised nude mice bearing subcutaneous xenografts of human cancer cell lines that had responded favorably to statin treatment in vitro (Fig. 1). When tumors became palpable at the subcutaneous injection sites of ES2 cells (p53 S241F/ S148F), mice were randomized and treated with 10 mg/kg Rosuvastatin or vehicle for 7 days a week (Fig. 2a). Tumors were measured every 3 days. On average, tumors on Rosuvastatin-treated mice grew slower (Fig. 2b) and were smaller at endpoint than their vehicle-treated counterparts (Fig. 2c).

To enhance the efficacy of statin treatment on xenografts, we repeated the experiment using MDA-MB-231 xenografts and placed mice on an isoprenoid-free diet (Ensure^R^)^26^, administered higher and more frequent doses of a more potent statin (Pitavastatin)^26^ and added Zoledronic Acid as a second mevalonate pathway inhibitor^25^ (Fig. 2d). Control mice were treated with both drug vehicles and also received the isoprenoid-free diet. Pitavastatin and Zoledronate treatment slowed mutp53 tumor growth and in some cases resulted in early, transient shrinkage (Fig. 2e). At endpoint, drug-treated tumors were smaller on average than vehicle-treated tumors (Fig. 2f). When protein lysates from these tumors were analyzed, the tumors that responded the most favorably to statin treatment had the lowest levels of mutant p53 (Fig. 2g), supporting that the antitumor effect of statins correlates with destabilization of mutp53.

**Fig. 3:**
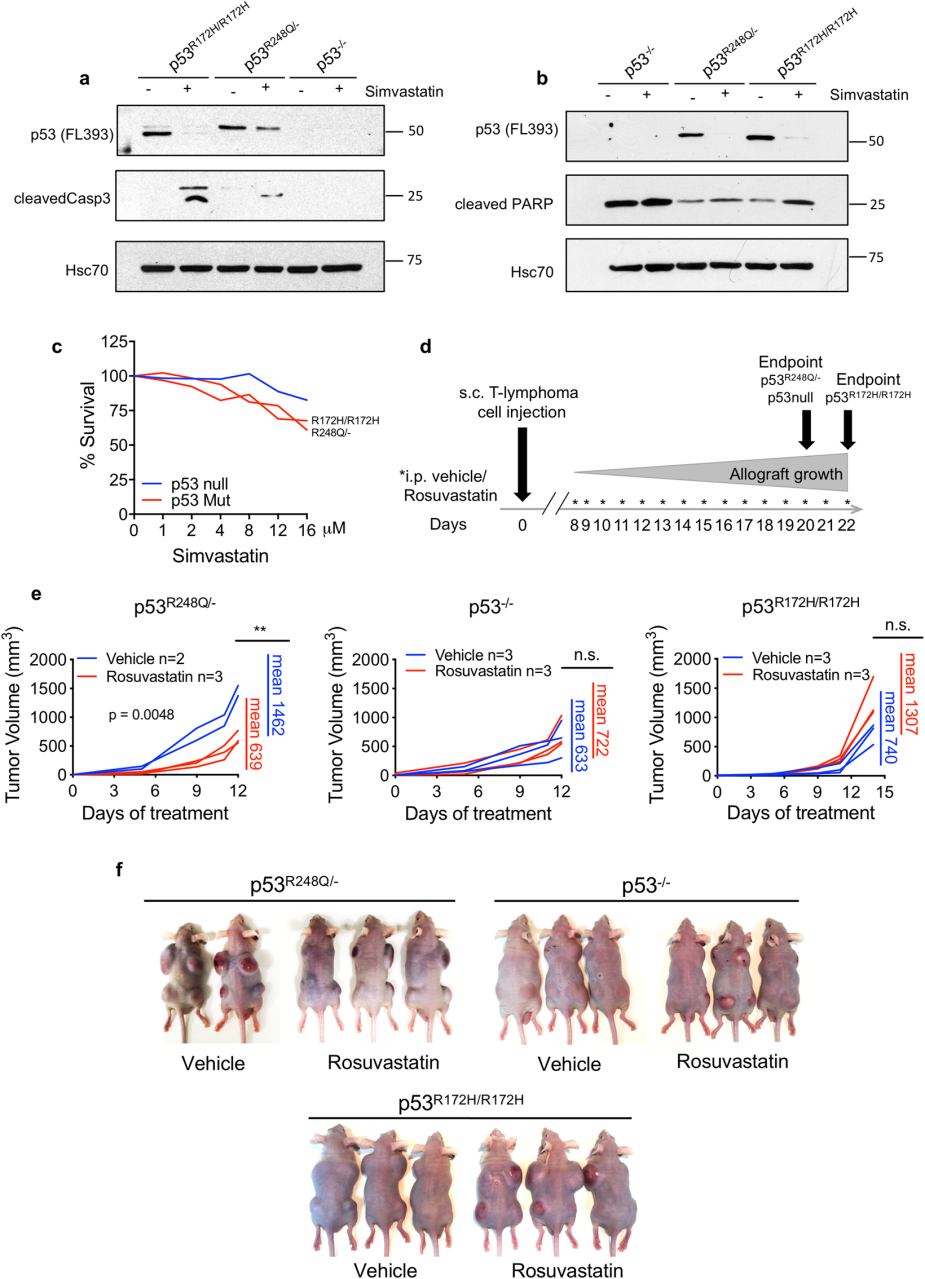
Statins downregulate GOF mutp53 and exhibit cytotoxic effects in murine T-lymphoma cell lines in vitro but have allele-selective anti-cancer effects in allografts. **a**, **b** Immunoblot analysis of T-lymphoma cell lines established from spontaneous tumors of mutp53 knockin mice p53^R248Q/−^, p53^R172H/R172H^, and p53 null littermate controls. Two independent experiments are shown. Cells were treated with 8 µM Simvastatin for 24 h. HSC70 as loading control. Blots were repeated from 3 to 5 independent experiments with similar results. **c** Dose response curve on the mouse lymphoma lines from **a**. Cells were treated with increasing doses of Simvastatin for 24 h. At endpoint the number of live cells was quantified by CellTiter Blue assay. **d**–**f** Rosuvastatin attenuates tumor growth allele-specifically in subcutaneous p53^R248Q/−^ but not in p53^R172H/R172H^ allograft tumors. **d** Timeline of the experiment. Immunocompromised nude mice were subcutaneously injected with 0.5 million murine T-lymphoma cells of the indicated genotypes per site in four dorsal locations (day 0). When palpable tumors began to form on day 8, mice were randomized and treated daily with vehicle or 10 mg/kg Rosuvastatin intraperitoneally. Tumors were measured frequently until day 20 when p53^R248Q/−^ and p53 null mice were killed (day 12 of treatment). p53^R172H^ mice were killed on day 22 (day 14 of treatment). **e** Quantitation of tumor growth (non-normalized) per mouse over time. Each line represents the average of all 4 tumors per mouse. Mean tumor volumes per mouse at endpoint (mean of Rosuvastatin-treated tumors per mouse vs. mean of vehicle-treated tumors per mouse) were compared between drug-treated and vehicle-treated mice. Unpaired Student’s *t*-test, **p < 0.01, n.s. nonsignificant. **f** Tumor burden at endpoint for all mice.

The observation of statin-induced mutp53 destabilization correlating with preferential cytotoxic effects in mutp53 human cancer cells was reproducible in murine tumor lines established from T-lymphomas of GOF knockin mice with genotypes p53^R248Q/−^ (humanized allele, corresponding to murine R245Q^27^) and p53^R172H/R172H^. Compared to p53^−/−^ littermate controls, both lymphoma lines responded to Simvastatin with mutp53 destabilization and simultaneous induction of cleaved caspase-3 and cleaved PARP (Fig. 3a, b). Cell viability assays confirmed the dose-dependent cytotoxic effect of Simvastatin in both lines, while p53 null cells were less affected (Fig. 3c). To determine in vivo efficacy, we treated subcutaneous allografts of these lymphoma lines. When palpable tumors began to form on day 8 (after injecting 0.5 million cells per site), animals of each genotype (p53 null, p53^R248Q/−^, and p53^R172H/R172H^) were randomized and treated with 10 mg/kg Rosuvastatin or vehicle i.p. for 7 days a week. Tumors were measured frequently until endpoint (Fig. 3d). While Rosuvastatin failed in p53 null allografts, it markedly attenuated tumor growth in p53^R248Q/−^ tumors by 2.3-fold compared to vehicle (Fig. 3e, f). Surprisingly, however, it had no effect on p53^R172H/R172H^ tumors. This is despite the in vitro efficacy of Simvastatin in these same murine cells (Fig. 3c) and in human cancer lines carrying the corresponding p53^R175H^ mutation (SkBr3 and TOV112D) (Fig. 1a–c).

**Fig. 4:**
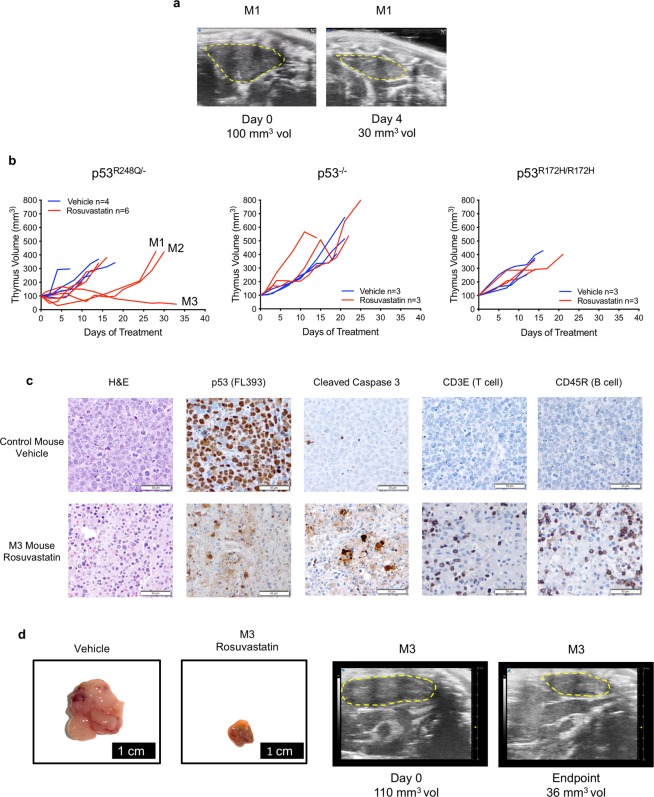
Statin monotherapy in GOF mutp53-driven autochthonous T-lymphoma has modest allele-selective effect. **a**, **b** Rosuvastatin causes transient tumor regression in p53^R248Q/−^ but not in p53^R172H/R172H^ mice with advanced autochthonous T-lymphoma. **a** The enlarged thymus of a p53^R248Q/−^ mouse (M1 in **b**) at treatment start and 4 days later when thymus volume had shrunk by 70%. Mice were monitored for thymus volume by three-dimensional ultrasound imaging, quantified by the Vevo Lab 3.1.0 Software. When tumors reached an average of 100 mm^3^ (ranging from 58 to 148 mm^3^), mice in each genotype group were randomized and treated with Rosuvastatin at 10 mg/kg or vehicle intraperitoneally for 5 days a week until moribund. **b** Quantitation of tumor growth over time. The initial tumor volume of each animal was normalized to 100 mm^3^. p53^R248Q/−^ mice show a transient decrease in tumor size to a variable degree (M1 versus M2) before tumor growth resumes. Mouse M3 responded by tumor stagnation followed by modest regression until endpoint. In contrast, p53^R172H/R172H^ and p53^−/−^ mice fail to respond to Rosuvastatin treatment**. c** Histology and immunohistochemistry of thymic tissue isolated from a vehicle-treated control mouse and the M3 mouse from **b** at endpoint. Representative images of each tissue are shown. The vehicle-treated lymphoma consists of dense sheets of monotonous malignant lymphoid cells exhibiting a high mitotic rate, a low background level of apoptosis and a complete absence of normal lymphocytes. Statin treatment resulted in prolonged stagnation and eventually modest shrinkage of the thymic lymphoma, histologically characterized by the tumor undergoing diffuse fibrosis with a marked diffuse inflammatory infiltrate consisting of numerous normal T- and B- cell lymphocytes, macrophages, and some neutrophils, preventing the clear identification of tumor cells. The apoptotic activity is elevated. H&E staining and immunostaining as indicated. Note that the few prominent brown cells in the M3 p53 immunostaining image are nonspecific macrophages showing cytoplasmic background myeloperoxidase staining. Moreover, vehicle tumors are devoid of normal T- and B- lymphocytes. All scale bars, 50 μm. **d** Visualization of the thymic tumor response from the two animals shown in **c**. Left, gross images of tumors; right, ultrasound imaging of the M3 tumor at beginning and endpoint of Rosuvastatin treatment.

Finally, to test the therapeutic anti-cancer effect of statins in an autochthonous in vivo setting of spontaneously developed mutp53-driven T-lymphomas, treatment of p53^R248Q/−^, p53^R172H/R172H^, and p53^−/−^ mice was started when mice had developed an enlarged thymus averaging 100 mm^3^ (ranging from 58 to 148 mm^3^) as determined by 3D ultrasound imaging. Mice were treated with 10 mg/kg Rosuvastatin (mimicking clinically achievable concentrations) or vehicle i.p for 5 days a week until the moribundity endpoint criteria of our animal protocol was reached. Thymus volume was closely monitored by frequent ultrasound imaging and quantified by the Vevo Lab 3.1.0 Software until endpoint. One of the p53^R248Q/−^ mice responded dramatically to Rosuvastatin with rapid tumor shrinkage by 70% over 4 days. However, the response was not durable since 14 days later the tumor had regained its initial volume and continued to grow robustly until moribundity (Fig. 4a, b; mouse M1). The second p53^R248Q/−^ mouse also transiently responded, albeit with a more modest decrease in tumor size and a shorter duration (Fig. 4b, mouse M2). A third mouse (M3) responded to statin-treatment by prolonged stagnation and then modest shrinkage until endpoint, which in this animal was determined by moribundity caused by an unrelated sarcoma (Fig. 4b, mouse M3). In the M3 mouse (Fig. 4d), statin treatment resulted in prolonged stagnation and shrinkage of the thymic lymphoma, histologically associated with diffuse fibrosis and a marked diffuse inflammatory infiltrate consisting of numerous normal T- and B-lymphocytes, macrophages and some neutrophils, obscuring clearly identifiable tumor cells. The apoptotic activity is elevated (Fig. 4c). The remaining three p53^R248Q/−^ mice failed to respond to statin treatment. Vehicle-treated control p53^R248Q/−^ mice also failed to show any tumor shrinkage. Similarly, the p53^−/−^ control mice failed to show any Rosuvastatin response. And notably, as already seen with allograft tumors (Fig. 3e), p53^R172H/R172H^ mice again failed to respond to Rosuvastatin (Fig. 4b).

## Discussion

The mevalonate pathway is a central metabolic hub that is essential for cellular cholesterol synthesis, membrane building, and intracellular transport, and as source of biologically active isoprenoids enabling lipid modification of numerous proteins for membrane association and cellular signaling. Its deregulation plays a role in transformation. Dysregulation of the mevalonate pathway by overexpressing HMGCR directly promotes cell transformation and cooperates with oncogenic signaling such as Ras and RhoA^28^.

A significant component of the multi-pronged tumorigenic activities of mutp53 GOF is the ability of stabilized mutp53 proteins to upregulate the mevalonate pathway. This was first identified in mutp53-expressing breast cancer cells in vitro where the ability of mutp53 to maintain a malignant morphologic phenotype in 3D cultures was mediated by this pathway. Direct inhibition of either HMGCR or any of the numerous downstream enzymes was as efficient in reverting their malignant invasive phenotype as was knockdown of mutp53 which caused downregulation of the enzymatic cascade^29^. Mechanistically, mutp53 is recruited to promoters of mevalonate pathway enzymes by functionally interacting with SREBP1/2 transcription factors and upregulating their transcriptional output. Of note, mutp53 status correlated with upregulation of 11 out of 17 mevalonate pathway genes in human breast cancer patients (cohort of 728 patients), and the subset of patients with the highest expression levels had the poorest prognosis^29^. Importantly, at the same time the mevalonate pathway also promotes the accumulation of mutp53 in cancer cells, suggesting the existence of a positive feedback loop via RhoA-mediated mechanical extracellular-to-cytoskeletal signaling^25^. Statins reactivate the Mdm2-dependent degradation of mutp53 by disrupting the protective mutp53-Hsp90 complex via two mechanisms. Inhibiting pathway flux with statins or preventing RhoA geranyl-geranylation with zoledronic acid or inhibitors of geranylgeranyl transferase type I impaired mutp53 stabilization in cancer cells^21,25^. Moreover, statins directly inhibit HDAC6 activity, leading to Hsp90 acetylation and inactivation and consequently mutp53 destabilization^24,25^. Thus, altogether there exists a significant molecular rationale for specifically exploring the statin family of HMGCR inhibitors as anticancer agents in mutp53 cancers.

We find that human and murine cancer cell lines in vitro show a reasonable although not strict correlation between Simvastatin and Atorvastatin-induced downregulation of mutp53 levels and concomitant apoptosis and cytotoxicity (Figs. 1 and 3a–c). We also find that in vivo Rosuvastatin monotherapy shows a modest anti-tumoral affect in human xenografts (Fig. 2a–c). In subsequent xenograft experiments (Fig. 2d–g), we attempted to enhance the statin effect by adding Zoledronic Acid (Zoledronate), which inhibits Farnesyl Pyrophosphate Synthase, a downstream enzyme of the mevalonate pathway^25^. Zoledronic acid has been shown to phenocopy the effects of statins on mutant p53 in vitro, and reduce xenograft growth and significantly decrease mutp53 accumulation in these tumors in vivo^25^. In addition, we followed steps suggested by Abdullah et al.^26^ in which they explore variables that might influence anticancer statin studies to explain the inconclusive results between these studies. These authors reported that standard mouse chow suppresses their anticancer effect^26^. One of the putative anti-cancer statin effects is the decreased production of isoprenoids which are essential for the post-translational modification of small GTPase oncogenes (e.g. Ras, Rac, and Rho)^26^. Literature data suggests the presence of exogenous isoprenoids such as geranylgeraniol present in standard mouse chow, thereby interfering with the statin effect^26^. Moreover, Abdullah et al.^26^ report that lipophilic statins have more potent anticancer effects than hydrophilic statins, and that both higher dosing and continual dosing (once every half-life) are required for statins to exert a significant antitumor effect. Thus, we replaced normal chow with Ensure^R^ plus (a liquid human diet supplement lacking isoprenoids) ad libitum, administered Pitavastatin (a lipophilic statin) at high concentration (59 mg/kg every 12 h), and added Zoledronic acid (200 μg/kg) every 3 days. This new treatment regimen successfully slowed or prevented tumor growth and did so to an overall better degree than Rosuvastatin monotherapy (compare Fig. 2b, e).

Rosuvastatin monotherapy shows a modest, allele-selective, and transient anti-tumoral effect in allograft and autochthonous T-lymphomas carrying the GOF p53 R248Q DNA contact mutation, compared to p53 null control mice (Figs. 3d–f and 4). This effect is variable, however, as not all p53 R248Q thymic tumors responded favorably to Rosuvastatin. However, encouragingly the tumor of one animal (mouse M3) underwent an impressive inflammatory-fibrosing response with little if any detectable remaining tumors cells. On the other hand, Rosuvastatin showed no effect in corresponding T-lymphomas expressing the GOF p53 R172H conformational mutant.

Thus, there is allele-dependent primary and acquired (given the transient effect) statin resistance and for clinical application it will be important in the future to identify its mechanisms especially in mutp53 solid cancers. Interestingly, in multiple myeloma (MM), a key element of tumor cell sensitivity to statin-induced apoptosis was found to lie in the normal feedback response of the mevalonate pathway to statins^28^. In MM, dysregulation of the classic feedback regulation is a key determinant of sensitivity. In sensitive cells, the feedback response to statin exposure is lost, resulting in a deficient compensatory upregulation of the rate-limiting HMGCR and its isoform HMGCS1 (hydroxymethylglutaryl coenzyme A synthase 1). In contrast, the statin-resistant MM lines overcame drug action by transcriptionally upregulating these key enzymes 3–10 fold in response to lovastatin exposure^28^.

Notably, allografts correctly predicted the allele-selective sensitivity of R248Q but not R172H tumors for the corresponding autochthonous T-lymphomas (compare Figs. 3e and 4b). However, in vitro the statin sensitivity of the same allelic system, at least as 2D cultures on plastic, was not a predictor for in vivo selectivity since both mutp53 alleles showed similar modest activity (compare Figs. 3c, e and 4b), rendering translational studies more cumbersome. Our finding that tumors expressing the DNA contact mutant R248Q showed T-lymphoma shrinkage/slow down while the conformational mutant R172H failed to respond to Rosuvastatin directly echoes that of Turrell et al. These authors also reported mutp53 allele specificity for Simvastatin sensitivity in that lung tumors driven by DNA contact mutant p53R270H;KrasG12D showed tumor shrinkage with a trend towards better short-term survival, while the corresponding lung tumors with conformational mutant p53R172H;KrasG12D failed to respond^30^. Xu et al. also found that the effects of statin on mutp53 vary in an allele-dependent manner. When tested on a panel of human pancreatic cancer cell lines in vitro, cells bearing the p53 conformational mutants R172H, I255N, G245S, and Y220C responded to statins, while cells bearing the p53 contact mutants R273H and R248W did not^11^. These findings together with ours suggests that potential statin therapy for mutp53 tumors will have to take this allele-specificity into account.

In sum, given the vast long-term clinical experience in the cardiovascular field, the low toxicity and low cost of this class of drugs, our results together with other studies discussed here are promising enough to justify future explorations of statins in combination with chemo- and targeted therapies in order to improve its anti-tumoral effect and better define the most statin-sensitive alleles and tumor types among mutp53-stabilized cancers.

## Materials and methods

### Human cell lines

The panel of mutp53 cell lines were breast adenocarcinoma SKBr3 (p53 R175H), MDAMB231 (p53 R280K), and BT549 (p53 R249S), ovarian adenocarcinoma TOV112D (p53 R175H), and ovarian clear cell carcinoma ES2 (p53 S241F/S148F), colorectal adenocarcinoma SW480 (p53 R275H/P309S). Control cells were breast adenocarcinoma MCF7 (wildtype p53), colorectal carcinoma HCT116 (p53 null) and bronchioalveolar carcinoma H358 (p53 null). All lines were maintained in Dulbecco’s modified Eagle’s medium (DMEM) with 10% fetal bovine serum, 1% penicillin-streptomycin, and 1% antibiotic-antimycotic. For this study STR-validated (each by 8 core loci) human cell lines were acquired from ATCC (Manassas, VA, USA). Cell lines were periodically tested every 3 months for mycoplasma using LookOut Mycoplasma PCR Detection Kit from Sigma-Aldrich (St. Louis, MO, USA) (MP0035-1KT).

### Chemicals and compounds

Atorvastatin and Rosuvastatin calcium salt were purchased from Cayman Chemicals (Ann Arbor, MI, USA). Simvastatin and Zoledronic Acid were purchased from Sigma-Aldrich. Pitavastatin was purchased from Adooq Bioscience (Irvine, CA, USA).

### Drug treatment

For in vitro studies, Atorvastatin and Simvastatin were dissolved in dimethyl sulfoxide. Pitavastatin was dissolved in 0.5% carboxymethyl cellulose.

For in vivo studies, Rosuvastatin was used because of its water solubility which avoids vehicle and diluent toxicity. Rosuvastatin was freshly dissolved in phosphate-buffered saline and injected intraperitoneally at 10 mg/kg for 7 days a week in the allograft experiments and for 5 days a week in the autochthonous lymphoma experiments.

For xenografts, Zoledronic Acid was dissolved in phosphate-buffered saline and injected intraperitoneally at 200 μg/kg every 3 days.

### Immunoblotting

Cells and tumor tissues were lysed with 0.5% TritonX100 in PBS (or TrisHCl, NaCl, glycerol, 1% Tritonx100, NaVO4, SDS) containing protease inhibitor cocktail (Sigma-Aldrich). Cell lysates (24–40 µg protein per lane) were separated by electrophoresis in 8–10% SDS PAGE gels, transferred to nitrocellulose membranes, blotted with primary antibodies, and appropriate secondary antibodies conjugated with HRP. The following antibodies were used: p53 (sc-126, clone DO1, 1:1000; sc-6243, FL393, 1:1000 from Santa Cruz Biotechnology, Dallas, TX, USA), cleaved caspase-3 (Asp175 clone 5A1E # 9664, 1:1000 from Cell Signaling, Danvers, MA, USA), cleaved PARP1 (clone SP276, ab225715, 1:1000 from Abcam, Cambridge, UK), β-actin (Abcam ab8227, 1:1000), and Hsc70 (clone 1B5, 1:1000 from Enzo Biochem, New York, NY, USA). Blots were developed with Clarity Western ECL Substrate (Bio-Rad Laboratories, Hercules, CA) on Premium Autoradiography Film (Denville Scientific, Metuchen, NJ, USA). Representative results of several different experiments are shown: for Fig. 1a, Simvastatin or Atorvastatin treatment of human cell lines were repeated 10–20 times on multiple different cell lines with different drug concentrations (biological replicates). The representative blot in Fig. 1a was repeated in three independent experiments with similar results (biological replicates). For Fig. 2g, tumor lysates were immunoblotted twice with similar results (technical replicates). For Fig. 3a and b, blots were repeated from 3 to 5 independent experiments with similar results (biological replicates).

### Immunohistochemistry

Tissues were fixed in formalin, embedded in paraffin and sectioned (3 µm). Slides were deparaffinized and boiled in Citric Acid Based Antigen Unmasking Solution from Vector Laboratories (Burlingame, CA, USA) (Cat # H-3300) for antigen retrieval, blocked in 10% goat serum and incubated overnight with the following primary antibodies: Santa Cruz FL393, Cat # sc-6243, 1:300; Cell Signaling cleaved caspase-3, Cat # 9661, 1:300; Invitrogen (Carlsbad, CA, USA) CD3e, Prod # MA5-14524, 1:500; BD Biosciences (San Diego, CA, USA) CD45R, Cat # 550286, 1:500). After PBS washing, slides were incubated either with biotinylated secondary antibody and VECTASTAIN Elite ABC Reagent from Vector Laboratories (Cat # PK-7100) or with ImmPRESS HRP Reagent from Vector Laboratories (Cat # MP-7404). They were then stained with DAB substrate with hematoxylin counterstain and coverslipped. Representative images were captured at ×40. All pictures for Fig. 4c were taken at the same time with the same microscope settings. Slides were imaged using Olympus Microscope BX41 and images were acquired using Olympus DP72 Microscope Digital Camera and Olympus cellSens Imaging Software, all from Olympus Corporation (Tokyo, Japan). All photos were processed using maximize contrast, adjust intensity (for brightening), and sharpen tools. No other manipulations were done. All scale bars, 50 μm.

### Cell viability assays

For CellTiterBlue Viability Assays (Promega, Madison, WI, USA), human cell lines were treated with increasing concentrations of Atorvastatin or Simvastatin for 48 h, then incubated at 37 °C with 20 µl of CellTiter-Blue reagent per 100 µl medium for 3 h until color changes from blue to pink, quantitated by multi-mode plate reader (FilterMax F5, Molecular Devices, San Jose, CA, USA). For Fig. 1b, two independent experiments were run with similar results (biological replicates). Mouse cell lines were treated for 24 h. For Fig. 3c, experiment was repeated three times (biological replicates). For cell counting, cell lines were treated with 32 µM Simvastatin for 48 h and then counted in Cellometer Cell Counting Chambers (SD100) using the Cellometer Vision Cell Profiler, both from Nexcelom Bioscience (Lawrence, MA, USA). For Fig. 1c, two independent experiments were run with similar results (biological replicates).

### Murine lymphoma cell lines and allograft transplants

Isolation of primary T-lymphoma cells was previously described^15^. Lymphoma cells were grown in Lymphoma Medium (1:1 DMEM/IMDM supplemented with 10% FBS, l-glutamine, penicillin–streptomycin, and β-mercaptoethanol, and passed through a 0.22-µm SteriCup and Steritop Vacuum Filtration System (Millipore Sigma, Burlington, MA, USA). Cells were immediately injected subcutaneously into immunocompromised nude mice at four dorsal sites per mouse with 0.5 million cells suspended per site in 3:1 PBS/Matrigel per site. Athymic nude mice (6–7-week-old Nu/Nu males) were acquired from Envigo (Indianapolis, IN, USA). In all, 18 mice were randomized into three genotype groups with six mice per genotype of allograft injection (p53^R248Q/−^, p53^R172H/R172H^, or p53 null). When palpable bumps began to appear at the injection sites, they were measured and treatment was begun. For treatment, mice were randomly assigned into drug and vehicle-treated groups, three mice per group. Tumor size was repeatedly measured and tumor volume calculated using the equation *V* = (*l* × *w* × *h*)/2. For each mouse, tumor size at all four injection sites was averaged and plotted over time. Mice were killed at protocol-allowed endpoint (2 cm^3^ tumors). p53^R248Q/−^ mice and p53^−/−^ mice were both killed at day 12, while p53^R172H/R172H^ mice were killed at day 14 after treatment start because no differential between vehicle and drug was seen at day 12. According to pre-established criteria, one mouse in the p53^R248Q/−^ vehicle group was excluded from the experiment because it failed to develop tumors.

### Xenografts

ES2 or MDA-MB-231 human cell lines were injected subcutaneously into immunocompromised nude mice at four dorsal sites per mouse with one million cells per site suspended in 3:1 PBS/Matrigel per site. Athymic nude mice (6–7 week old Nu/Nu males) were acquired from Envigo. When palpable bumps began to appear at the injection sites, they were measured and treatment was begun. For the ES2 xenograft, five mice were randomly assigned into two groups: drug-treated (3) and vehicle-treated (2). Treatment for ES2 xenografts consisted of intraperitoneal injections of Rosuvastatin at 10 mg/kg for 7 days a week. For MDA-MB-231 xenografts, on the day of the xenograft injection, standard mouse chow was removed and replaced with Ensure plus, which was provided ad libitum until endpoint. Four mice were randomly assigned to two groups: drug-treated and vehicle-treated. Treatment consisted of oral administration (gavage) of Pitavastatin at 59 mg/kg every 12 h, with intraperitoneal injections of Zoledronic Acid at 200 μg/kg every 3 days. According to pre-established criteria, one mouse was excluded from the vehicle group because it failed to develop tumors.

For both experiments, tumor size was repeatedly measured and tumor volume calculated using the equation *V* = (*l* × *w* × *h*)/2. In the case of the ES2 xenografts, average initial tumor volume was normalized to 40 mm^3^. In the case of the MDA-MB-231 xenografts, volume measurements of each tumor were normalized to an initial volume of 20 mm^3^. Mice were killed at protocol-allowed endpoint (2 cm^3^ tumors). ES2 xenograft mice were killed on day 23 (day 16 of treatment). MDA-MB-231 xenograft mice were killed on day 45 (day 30 of treatment). For MDA-MB-231 xenografts, pieces of tumor were collected, snap-frozen, ground in liquid nitrogen with a mortar and pestle, and resuspended in a lysis buffer consisting of TrisHCl, NaCl, glycerol, 1% Tritonx100, NaVO4, SDS, and protease inhibitor cocktail. Lysate was sonicated and quantified using Bradford Assay.

### High-resolution ultrasound imaging

Hotspot humanized p53 R248Q/− mice and p53 R172H/R172H mice as well as p53−/− controls (all C57/Bl6J background) were previously described^15^. Parental p53 R248Q/+ and p53−/+ strains on were crossed to obtain p53 R248Q/− and p53−/− mice. p53 R172H/R172H mice were generated by interbreeding R172H/+ mice. Both male and female mice were used, ranging in age from 2 months to 1 year. Animals were regularly monitored by ultrasound imaging for development of thymic tumors using the Visual Sonics Vevo 3100 High-Resolution Imaging System from FUJUFILM Visual Sonics (Toronto, Canada). When tumors had reached 100 mm^3^ on average (ranging from 58 to 148 mm^3^), treatment was initiated. Mice were assigned randomly to their experimental group at the start of treatment. For the p53^R248Q/−^ group, 4 mice were treated with vehicle and 6 mice were treated with Rosuvastatin. For the p53 null group, three mice were treated with vehicle and three mice were treated with Rosuvastatin. For the p53^R172H/R172H^ group, three mice were treated with vehicle and three mice were treated with Rosuvastatin. Intraperitoneal Rosuvastatin treatment was given five times a week accompanied by repeated ultrasound imaging of the thymus using a mobile probe to reconstruct 3D tumor volumes by stacking serial slices with the Vevo Lab 3.1.0 software (FUJIFILM VisualSonics). All tumor measurements were normalized to an initial volume of 100 mm^3^ and plotted over time. Animals were treated humanely according to the guidelines issued by the Institutional Animal Care and Use Committee at Stony Brook University and were killed when reaching the endpoint of moribundity.

### Radiation

Due to their genotypes, our p53 mutant and null mice develop T-lymphomas spontaneously, but in some cases mice were given a one-time radiation dose of 6 Gy in order to accelerate the development of thymic tumors.

### Statistical and general methods

Error bars represent s.d. Center values represent the mean. Unpaired Student’s *t*-tests were used to determine statistical significance, defined as *p* < 0.05. For Fig. 1c, the variance between the groups that were statistically being compared was similar. For Figs. 1b, 2b, e, and 3c, e, control vs experimental cell lines and animals (blue vs red), the endpoint values were compared for significant difference. No statistical methods were used to pre-determine sample size. Researchers were not blinded during data collection or assessment of outcome.
